# lncRNA KCNQ1OT1 reverses the effect of sevoflurane on hepatocellular carcinoma progression via regulating the miR-29a-3p/CBX3 axis

**DOI:** 10.1590/1414-431X2020e10213

**Published:** 2021-05-17

**Authors:** Weifu Zhou, Hui Li, Shuo Shang, Feng Liu

**Affiliations:** 1 Department of Anesthesiology, Zhangqiu District People’s Hospital, Jinan, Shandong, China; 2 Department of Anesthesiology, Zhangqiu Maternal and Child Health Hospital, Jinan, Shandong, China; 3 Department of Anesthesiology, the First Hospital of Yulin, Yulin, Shaanxi, China

**Keywords:** Sevoflurane, Hepatocellular carcinoma, KCNQ1OT1, miR-29a-3p, CBX3

## Abstract

Sevoflurane (SEVO) is widely applied as an anesthetic, which exerts antitumor capacity in various cancers, including hepatocellular carcinoma (HCC). Previous studies indicated that long non-coding RNA KCNQ1 opposite strand/antisense transcript 1 (KCNQ1OT1) was upregulated, while microRNA-29a-3p (miR-29a-3p) was downregulated in HCC. Thus, we aimed to explore the roles of KCNQ1OT1 and miR-29a-3p in HCC cells exposed to SEVO. Cell proliferation, apoptosis, migration, and invasion were assessed by the 3-(4,5-dimethylthiazol-2-yl)-2,5-diphenyltetrazolium bromide (MTT) assay, flow cytometry, and transwell assays, respectively. The levels of genes were determined by quantitative real-time polymerase chain reaction (qRT-PCR) or western blot. Furthermore, the interaction between miR-29a-3p and KCNQ1OT1 or chromebox protein homolog 3 (CBX3) was predicted by Starbase or Targetscan, and then confirmed by dual-luciferase reporter assay. We found that the levels of KCNQ1OT1 and CBX3 were decreased, while miR-29a-3p was increased in SEVO-treated HCC cells. KCNQ1OT1 overexpression weakened the inhibitory effects of SEVO on HCC cell proliferation, apoptosis, migration, and invasion. Interestingly, KCNQ1OT1 bound to miR-29a-3p, and miR-29a-3p targeted CBX3. KCNQ1OT1 upregulated CBX3 level by repressing miR-29a-3p expression. Furthermore, KCNQ1OT1 exerted tumor promotion in HCC cells via suppressing miR-29a-3p to regulate CBX3 expression. Collectively, our findings demonstrated that KCNQ1OT1 regulated the antitumor effects of SEVO on HCC cells through modulating the miR-29a-3p/CBX3 axis, providing a theoretical basis for the treatment of HCC.

## Introduction

Hepatocellular carcinoma (HCC) is one of the major causes of cancer-associated death worldwide (1). A report showed that there were more than 466,100 new cases of HCC and 422,100 HCC-related deaths in China in 2015 (2). The survival rate for HCC patients is still low due to the high incidence of metastasis (3). Sevoflurane (SEVO), an anesthetic agent, is widely applied for clinical therapy of diseases and plays a suppressive role in various cancers (4). For instance, SEVO can inhibit cell proliferation and cell cycle in breast cancer (5). Moreover, SEVO acted as a suppressor in lung carcinoma progression (6). Furthermore, SEVO suppressed the development of HCC (7). However, the detailed mechanisms of SEVO in HCC cells remain unclear.

Long non-coding RNAs (lncRNAs), with over 200 nucleotides, are considered a group of conserved RNAs that regulate a variety of cell behaviors, including cell proliferation, mobility, and autophagy (8). lncRNA KCNQ1 opposite strand/antisense transcript 1 (KCNQ1OT1) was identified as an oncogene in human cancers. For example, Liu et al. (9) reported that KCNQ1OT1 elevated cell growth and metastasis and suppressed cell apoptosis in colorectal cancer. Moreover, KCNQ1OT1 level was elevated in HCC tissues and positively regulated HCC progression through regulating miR-504 expression (10). However, the functional mechanism of KCNQ1OT1 in HCC is not fully reported.

MicroRNAs (miRNAs), with approximately 20 nucleotides in length, modulate the levels of downstream genes through targeting 3′untranslated region (3′UTR) of mRNA in human cancers (11). For instance, microRNA-29a-3p (miR-29a-3p) inhibited cell proliferation in HCC (12). Furthermore, Xiao et al. (13) suggested that miR-29a-3p repressed HCC cell proliferation and mobility. Moreover, recent research indicates that SEVO exerted antitumor activity in HCC by regulating miR-29a expression (14). Starbase (http://starbase.sysu.edu.cn/) predicted that miR-29a-3p might be a target of KCNQ1OT1. Therefore, it is essential to investigate the functional mechanism of KCNQ1OT1 and miR-29a-3p in SEVO-treated HCC cells.

Chromebox protein homolog 3 (CBX3) was reported as an oncogene to positively mediate the development of many human cancers, such as pancreatic cancer (15), tongue squamous cell carcinoma (16), and osteosarcoma (17). Emerging evidence has shown that CBX3 enhances HCC cell proliferation and acts as a biomarker for the prognosis of HCC patients (18). Moreover, TargetScan (http://www.targetscan.org) predicted that CBX3 harbored the binding sites with miR-29a-3p. Thus, we explored the role of CBX3 in SEVO-treated HCC cells.

We hypothesized that there was the lncRNA/miRNA/mRNA regulatory axis in SEVO-treated HCC. Thus, the association among KCNQ1OT1, miR-29a-3p, and CBX3 was explored in SEVO-treated HCC cells.

## Material and Methods

### Tissues and cell culture

Thirty HCC tumor tissues were obtained from 30 HCC patients, and 30 HCC tumor-SEVO tissues were collected from 30 HCC patients treated with SEVO at the Sixth People's Hospital of Jinan. The clinicopathological features of these 60 HCC patients are presented in Table 1. This research was approved by the Ethics Review Committees of the Sixth People's Hospital of Jinan. All patients provided written informed contents.

**Table 1:** Clinicopathological features of the hepatocellular carcinoma patients.

Parameters	Numbers (n=60)
Age	
≤55	21 (35.0%)
>55	39 (65.0%)
Gender	
Male	42 (70.0%)
Female	18 (30.0%)
TNM stage	
I+II	27 (45.0%)
III+IV	33 (55.0%)
Tumor size	
≤4 cm	36 (60.0%)
>4 cm	24 (40.0%)
Metastasis	
Negative	32 (53.3%)
Positive	28 (46.7%)

TNM: tumor/node/metastasis.

Two HCC cell lines (Huh7 and Hep3B) were provided by the Chinese Academy of Sciences cell bank (China), and then incubated in Dulbecco's modified Eagle's medium (DMEM; Gibco, USA) at 37°C with 5% CO_2_. The medium was added with 10% fetal bovine serum (FBS; Gibco) and 1% v/v penicillin/streptomycin (Millipore, USA).

The experimental gas mixtures were 4% SEVO with 21% O_2_/5% CO_2_ balanced with nitrogen. Cells were placed in an airtight gas chamber, equipped with inlet and outlet valves. The gas was delivered at 6 L/min through calibrated vaporizers (Draeger, Germany). The chamber gases were monitored using an anesthetic analyzer (Datex-Ohmeda, UK). SEVO (Sigma, USA) at 4% was used to treat HCC cells for 48 h as previously described (14).

### Cell proliferation assay

Cell proliferation was examined using the 3-(4,5-dimethylthiazol-2-yl)-2,5-diphenyltetrazolium bromide (MTT) kit (Promega, USA) according to the manufacturer’s manual. In brief, 6×10^3^ cells were seeded into 96-well plates. After transfection and stimulation with SEVO, 20 μL MTT solution was added to each well and cultured for 4 h at different time points (0, 24, 48, 72 h). Then, cell supernatants were removed and 200 μL dimethyl sulfoxide (DMSO) was added to each well. Finally, the microplate reader (Bio-Rad, USA) was used to examine the absorbance of the samples at 490 nm.

### Cell apoptosis assay

The apoptosis of HCC cells was monitored by flow cytometry with an Annexin V-FITC/PI Apoptosis Detection Kit (Invitrogen, USA) following the manufacturer’s manual. After transfection and stimulation with SEVO, the cells were harvested, washed, and stained by 5 μL Annexin-V fluorescein isothiocyanate (FITC) and propidium iodide (PI) following the recommended protocol. Finally, a flow cytometer (BD Biosciences, USA) was used to analyze the cell apoptosis rate.

### Cell migration and invasion assay

Cell mobility was analyzed using a transwell chamber (Millipore) following the manufacturer’s recommendations. The insert was coated with matrigel (BD Bioscience) for the invasion assay, while an insert without matrigel was used for the migration assay. Briefly, treated or transfected cells were collected, re-suspended in 100 μL serum-free medium, and seeded into the upper chamber at a density of 8×10^4^ cells/well. The lower chamber was added with 500 μL of the corresponding medium with 10% FBS. After culture for 24 h, migratory or invasive cells were fixed, stained, and measured by a microscope (Olympus, Japan).

### RNA extraction and quantitative real-time polymerase chain reaction (qRT-PCR)

Total RNA from HCC tissues and cells was obtained using TRIzol (Invitrogen). Reversely transcription assay (5 μg RNA) was performed to synthesize complementary DNA (cDNA) using PrimeScript RT reagent kit (TaKaRa Bio, Japan) according to the manufacturer's protocol. In brief, transcription was conducted in a 10 μL reaction mixture, including polyadenylated RNA (100 ng), 5× PrimeScript Buffer (2 μL), PrimeScript RT Enzyme Mix I (0.5 μL), RT primer mixture (1 μL), and RNase-free water. Total reaction mixture was incubated at 50°C for 15 min and 85°C for 5 s. Then, SYBR Green Mix (Beyotime Biotechnology, China) was employed to carry out qRT-PCR. Final volumes (15 μL) contained 1.5 μL template cDNA mixed with 7.5 μL 2× SYBR Green PCR master mix, and 3 μL of each forward and reverse primers. The expression of miR-29a-3p, KCNQ1OT1, and CBX3 was calculated using the 2^-ΔΔCt^ method and normalized by the expression of U6 or glyceraldehyde 3-phosphate dehydrogenase (GAPDH). The amplification parameters were as follows: denaturation at 95°C for 10 min, followed by 40 cycles of denaturation at 95°C for 30 s, annealing at 60°C for 30 s, and extension at 72°C for 1 min. The primer sequences used in this study were: KCNQ1OT1 (forward (F), 5′-CTTTGCAGCAACCTCCTTGT-3′; reverse (R), 5′-TGGGGTGAGGGATCTGAA-3′); miR-29a-3p (F, 5′-AGCACCAUCUGAAAUCGGUUA-3′; R, 5′-GTGCAGGGTCCGAGGT-3′); CBX3 (F, 5′-GAGATGCTGCTGACAAACCA-3′; R, 5′- TATTTGCCTCTTTCGCCAGC-3′); U6 (F, 5′-TGCGGGTGCTCGCTTCGGCAGC-3′; R, 5′-CCAGTGCAGGGTCCGAGGT-3′; and GAPDH (F, 5′-ATCACTGCCACCCAGAAGAC-3′; R, 5′-TTTCTAGACGGCAGGTCAGG-3′).

### Cell transfection

miR-29a-3p mimic (miR-29a-3p, GeneBank accession No. NR_029503.1) and the control (miR-NC), miR-29a-3p inhibitor (anti-miR-29a-3p) and the negative control (anti-miR-NC), and small inferring RNA against CBX3 (si-CBX3: 5′-GCGTTTCTTAACTCTCAGAAA-3′) and the control (si-NC: 5′-TTCTCCGAACGTGTCACGT-3′) were purchased from Ribobio (China). One day before transfection, Huh7 and Hep3B cells were seeded into a 12-well plate. Cells (5×10^5^ cells/well) were transfected with 0.5 μg of the aforementioned oligonucleotides using 0.6 μL of Lipofectamine 2000 (Invitrogen). KCNQ1OT1 (GeneBank accession No. NR_002728) sequence was cloned into the pcDNA3.1 plasmid (Genepharma, China) to generate KCNQ1OT1 overexpression vector. Then, 0.2 μg of KCNQ1OT1 was transfected in Huh7 and Hep3B cells with 0.5 μL of Lipofectamine 2000 reagent (Invitrogen). After incubation for 48 h, transfected cells were collected for further experiments.

### Western blot assay

Cells were lysed by RIPA buffer (Beyotime Biotechnology) to isolate the proteins. Then, 30 μg were subjected to 10% sodium dodecyl sulfonate-polyacrylamide gel electrophoresis (SDS-PAGE), electro-transferred onto polyvinylidene difluoride (PVDF) membranes (Millipore), and blocked by 5% non-fat milk in phosphate-buffered solution (PBST) at room temperature for 1 h. Next, the membranes were incubated with the primary antibody against CBX3 (1:1,000; ab213167, Abcam, USA), B-cell lymphoma 2 (Bcl-2) (1:1,000; ab32124, Abcam), BCL2 associated X (Bax) (1:1,000; ab32503, Abcam), cleaved caspase3 (Cleaved-casp-3; 1:1,000; ab13847, Abcam), or β-actin (1:1,000; ab5694, Abcam) overnight at 4°C, and then incubated with the corresponding secondary antibody (1:2,000; ab150077, Abcam) for 1 h at 37°C. Finally, the proteins were visualized using ECL reagents (Millipore).

### Dual-luciferase reporter assay

Based on the bioinformatics prediction results, the wild-type or mutant-type HOXA10 3′UTR sequence and CBX3 3′UTR sequence containing miR-29a-3p binding sites (putative binding sequence: ACCACGA) were amplified and cloned into pmirGLO vector (Promega), termed as KCNQ1OT1-WT (putative binding sequence: TGGTGCT), KCNQ1OT1-MUT (mutant binding sequence: GTTGTAG), CBX3-WT (putative binding sequence: UGGUGCU), and CBX3-MUT (mutant binding sequence: GUUGUAG) reporter plasmids. Then, reporter plasmids were co-transfected with 400 ng of the constructed plasmids, 50 ng of renilla luciferase reporter plasmid (pRL-TK), and 50 nM of miR-29a-3p or miR-NC using Lipofectamine 2000 (Invitrogen). After incubation for 48 h, the luciferase density was determined by the dual luciferase assay system (Promega). Renilla luciferase activities were used as the internal control for the normalization of firefly luciferase activity.

### Statistical analysis

The data, from at least three independent experiments, are reported as means±SD. The difference between two groups or numerous groups were analyzed using Student's *t*-test or one-way analysis of variance (ANOVA). Spearman's correlation coefficient was used to investigate the association between the levels of two genes. P<0.05 was considered as significantly different.

## Results

### SEVO treatment suppressed cell proliferation, migration, invasion, and induced apoptosis of HCC cells

**Figure 1:**
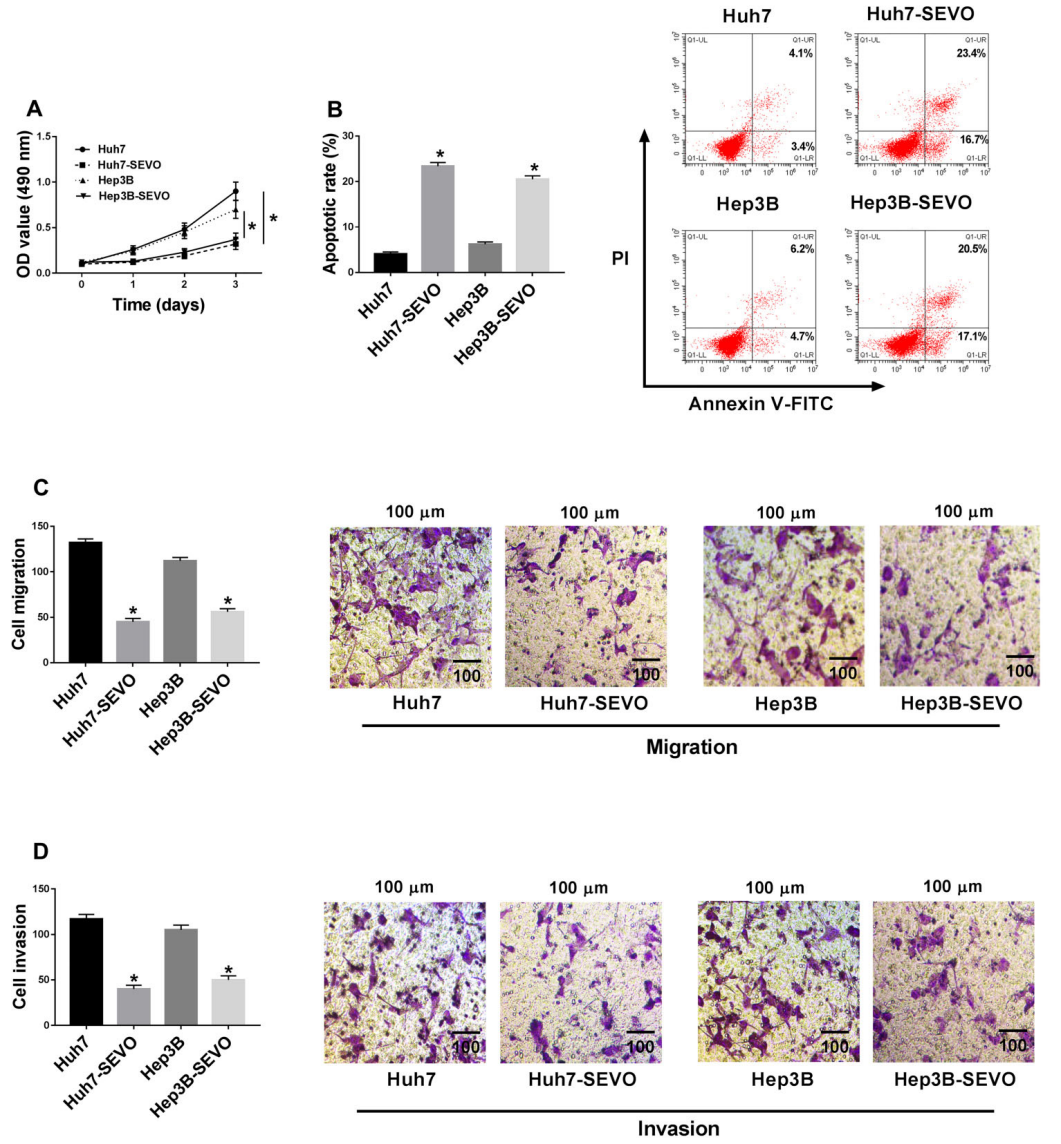
Sevoflurane (SEVO) suppressed cell proliferation, migration, and invasion and promoted cell apoptosis in hepatocellular carcinoma cells. Huh7 and Hep3B cells were stimulated with 4% SEVO for 48 h. **A**, MTT assay was performed to determine cell proliferation ability (n=3). **B**, Flow cytometry was used to analyze cell apoptosis rate (n=3). **C** and **D**, Cell migration and invasion were assessed by transwell assays (scale bar: 100 μm) (n=3). Data are reported as means±SD. *P<0.05 (Student's *t*-test).

As shown in Figure 1A, treatment with SEVO significantly suppressed cell proliferation. Flow cytometry analysis suggested that the cell apoptosis rate was significantly upregulated by SEVO in Huh7 and Hep3B cells (Figure 1B). Moreover, we found that cell migratory and invasive abilities were repressed by SEVO treatment (Figure 1C and D). These data confirmed that SEVO repressed HCC progression.

### KCNQ1OT1 overexpression weakened the effect of SEVO on HCC progression

**Figure 2:**
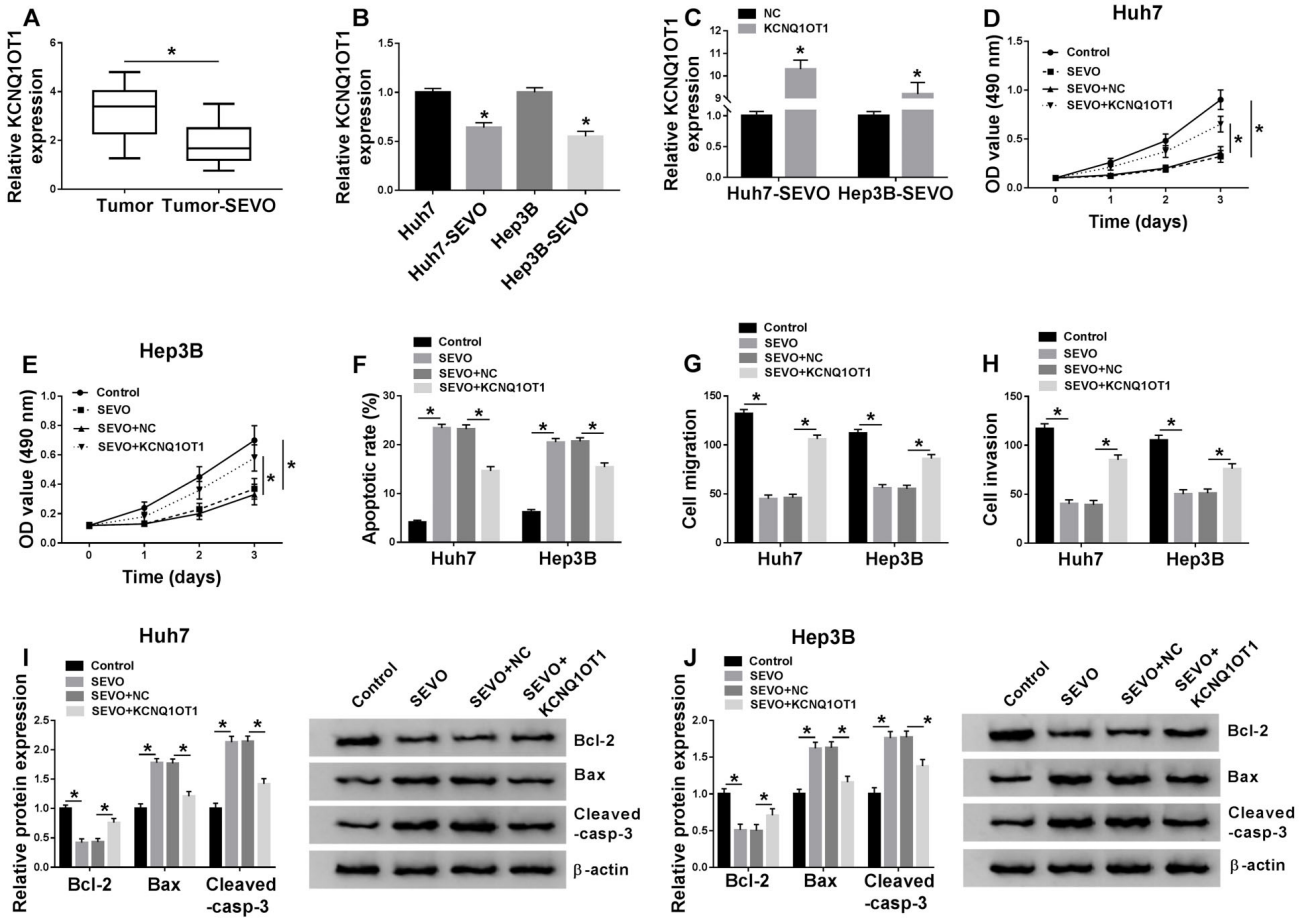
KCNQ1OT1 overexpression reversed the effects of sevoflurane (SEVO) on hepatocellular carcinoma (HCC) progression. Huh7 and Hep3B cells were transfected with KCNQ1OT1 or negative control (NC) and then stimulated with 4% SEVO for 48 h. **A**, KCNQ1OT1 expression was detected by qRT-PCR assay in HCC tumor tissues (n=30) and SEVO-treated HCC tumor tissues (n=30). Data are reported as median and interquartile range. *P<0.05 (Kruskal-Wallis). **B** and **C**, KCNQ1OT1 expression was detected by qRT-PCR assay (n=3). **D** and **E**, Cell proliferation ability was measured by MTT assay (n=3). **F**, Cell apoptosis rate was examined by flow cytometry (n=3). **G** and **H**, Transwell assay was employed to investigate cell migration and invasion (n=3). **I** and **J**, Western blot assay was carried out to measure the levels of cell apoptosis-related proteins (Bcl-2, Bax, and Cleaved-casp-3) (n=3). Data are reported as means±SD. *P<0.05 (Student's *t*-test or ANOVA).

KCNQ1OT1 level was downregulated in tumor-SEVO HCC tissues (Figure 2A). Moreover, decreased KCNQ1OT1 level was observed in SEVO-treated HCC cells (Huh7-SEVO and Hep3B-SEVO) (Figure 2B). To further explore whether KCNQ1OT1 affected the effects of SEVO on HCC progression, Huh7-SEVO and Hep3B-SEVO cells were transfected with NC or KCNQ1OT1. QRT-PCR assay suggested that transfection of KCNQ1OT1 overexpression vector significantly increased the expression of KCNQ1OT1 (Figure 2C). As demonstrated in Figure 2D and E, the inhibition effect of SEVO on cell proliferation was abolished by upregulation of KCNQ1OT1. Furthermore, we found that SEVO significantly induced cell apoptosis, whereas this action was weakened by KCNQ1OT1 overexpression (Figure 2F). Transwell migration and invasion assays confirmed that KCNQ1OT1 overexpression reversed the effect of SEVO on cell migration and invasion (Figure 2G and H). Moreover, we detected the expression of cell apoptosis-related proteins, including Bcl-2, Bax, and Cleaved-casp-3. Consistent with the results from flow cytometry, our data showed that SEVO decreased Bcl-2 expression, but enhanced Bax and Cleaved-casp-3 expressions in Huh7 and Hep3B cells, which were abolished by KCNQ1OT1 overexpression, suggesting that the promotion effect of SEVO on cell apoptosis was reversed by KCNQ1OT1 (Figure 2I and J). Therefore, SEVO inhibited HCC progression by downregulating KCNQ1OT1 expression.

### miR-29a-3p was a target of KCNQ1OT1

**Figure 3:**
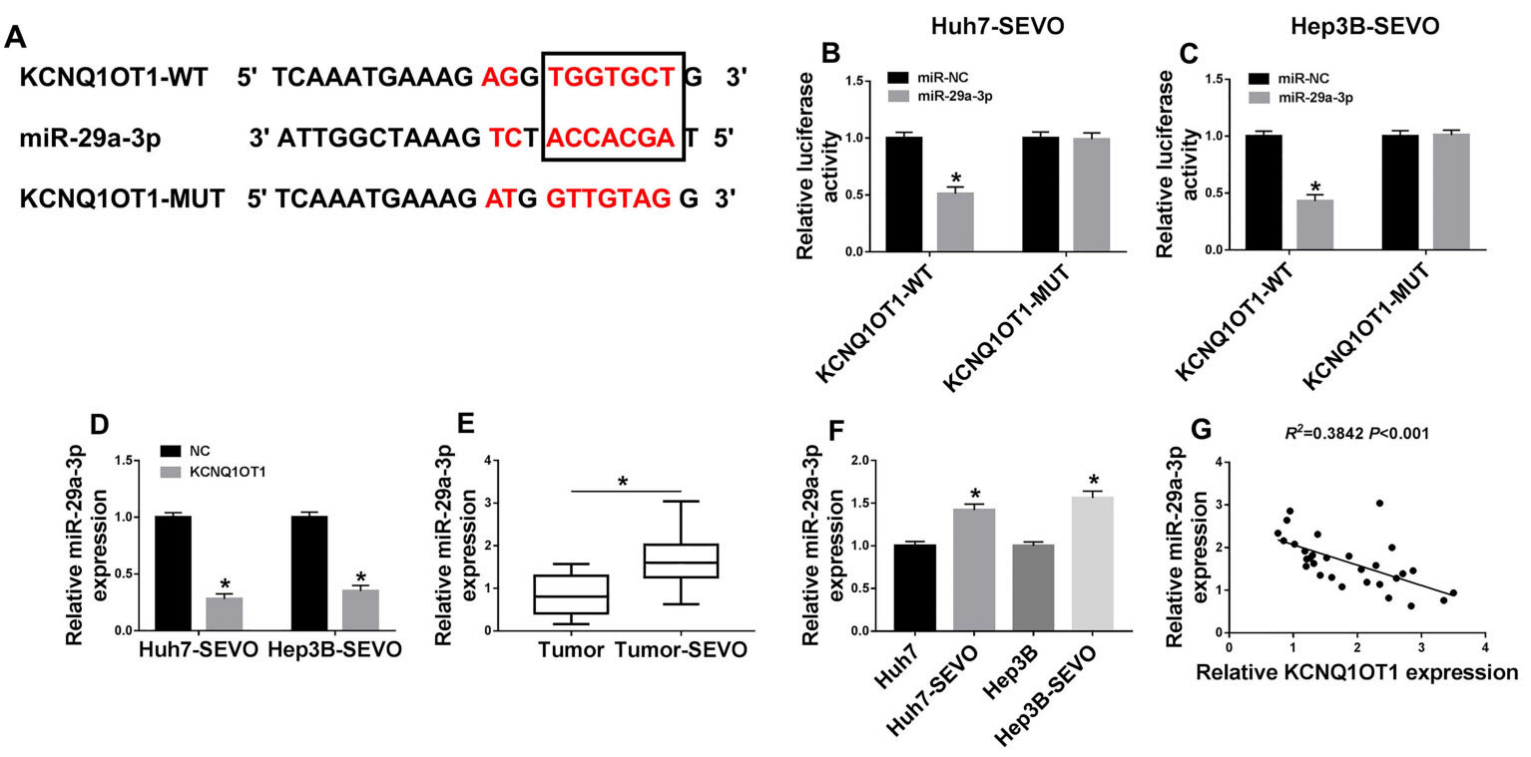
A, The interaction between KCNQ1OT1 and miR-29a-3p was predicted by online tool Starbase. **B** and **C**, Huh7 and Hep3B cells were treated with sevoflurane (SEVO) for 48 h. The luciferase activity of Huh7-SEVO and Hep3B-SEVO cells transfected with KCNQ1OT1-WT (wild-type) or KCNQ1OT1-MUT (mutant) and miR-29a-3p or miR-NC (negative control) was determined (n=3). **D**, miR-29a-3p level was detected by qRT-PCR assay in Huh7-SEVO and Hep3B-SEVO cells transfected with NC or KCNQ1OT1 (n=3). **E**, miR-29a-3p level was measured in hepatocellular carcinoma (HCC) tumor tissues (n=30) and SEVO-treated HCC tumor tissues (n=30). **F**, miR-29a-3p level was detected by qRT-PCR in Huh7 and Hep3B cells, as well as Huh7-SEVO and Hep3B-SEVO cells (n=3). **G**, The relationship between KCNQ1OT1 level and miR-29a-3p level was explored. Data are reported as means±SD. *P<0.05 (Student's *t*-test or ANOVA).

The online tool Starbase predicted that miR-29a-3p was a potential target gene of KCNQ1OT1 (Figure 3A). Then, dual-luciferase reporter assay was employed to verify this interaction. As shown in Figure 3B and C, miR-29a-3p reduced the luciferase activity in the KCNQ1OT1-WT group, not in the KCNQ1OT1-MUT group. Next, we analyzed the effect of KCNQ1OT1 on miR-29a-3p expression, and found that miR-29a-3p level was dramatically downregulated by KCNQ1OT1 overexpression, meaning that KCNQ1OT1 negatively regulated miR-29a-3p expression (Figure 3D). Furthermore, increased miR-29a-3p level was observed in SEVO-treated HCC tissues and cells (Figure 3E and F). Moreover, miR-29a-3p level was negatively correlated with KCNQ1OT1 level in SEVO-treated HCC tissues (Figure 3G). These results suggested that KCNQ1OT1 sponged miR-29a-3p and negatively regulated miR-29a-3p expression.

### miR-29a-3p knockdown reversed the effect of SEVO on HCC progression

**Figure 4:**
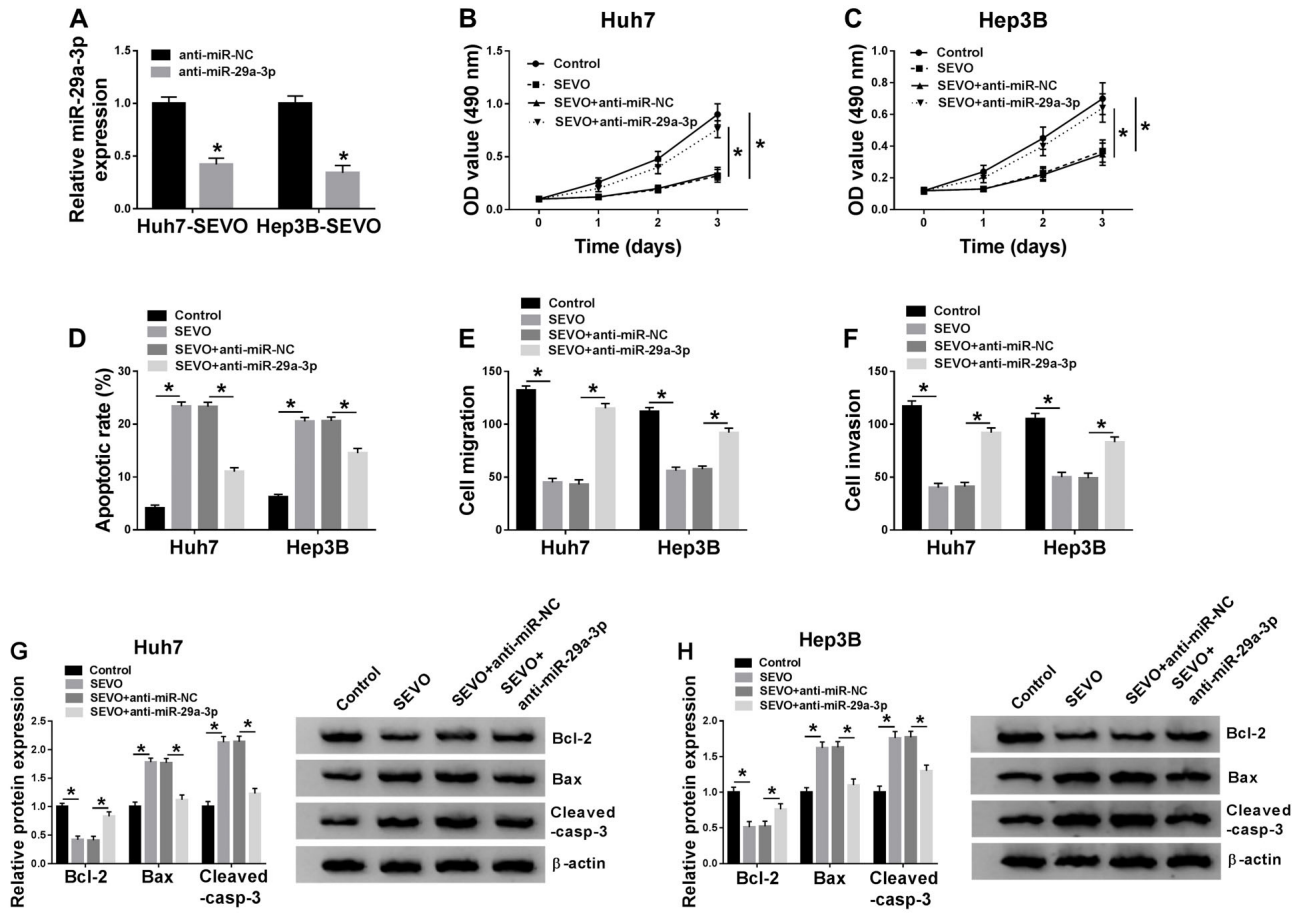
miR-29a-3p depletion blocked the effects of sevoflurane (SEVO) on hepatocellular carcinoma (HCC) progression. The Huh7 and Hep3B cells were transfected with anti-miR-29a-3p or anti-miR-NC (negative control) and then stimulated with 4% SEVO for 48 h. **A**, miR-29a-3p expression was examined by qRT-PCR (n=3). **B** and **C**, MTT assay was employed to assess cell proliferation ability (n=3). **D**, Flow cytometry was used to determine cell apoptosis rate (n=3). **E** and **F,** Transwell assay was employed to assess cell migration and invasion (n=3). **G** and **H**, The levels of cell apoptosis-related proteins were detected by western blot assay (n=3). Data are reported as means±SD. *P<0.05 (Student's *t*-test or ANOVA).

To investigate the effect of miR-29a-3p on HCC cells under SEVO treatment, Huh7-SEVO and Hep3B-SEVO cells were transfected with anti-miR-29a-3p or anti-miR-NC. qRT-PCR assay confirmed that the transfection with anti-miR-29a-3p significantly downregulated miR-29a-3p expression (Figure 4A). As shown in Figure 4B and C, cell proliferation was remarkably repressed by SEVO, which was rescued by miR-29a-3p inhibitor. Also, miR-29a-3p knockdown suppressed SEVO-induced cell apoptosis (Figure 4D). The results suggested that SEVO significantly inhibited cell migration and invasion, whereas this action was reversed by miR-29a-3p inhibitor (Figure 4E and F). Furthermore, we found that the promotion effects of SEVO on Bax and Cleaved-casp-3 expression as well as the inhibition effect on Bcl-2 expression were abolished by miR-29a-3p inhibitor (Figure 4G and H). Taken together, SEVO repressed HCC progression via upregulating miR-29a-3p expression.

### miR-29a-3p targeted CBX3

**Figure 5:**
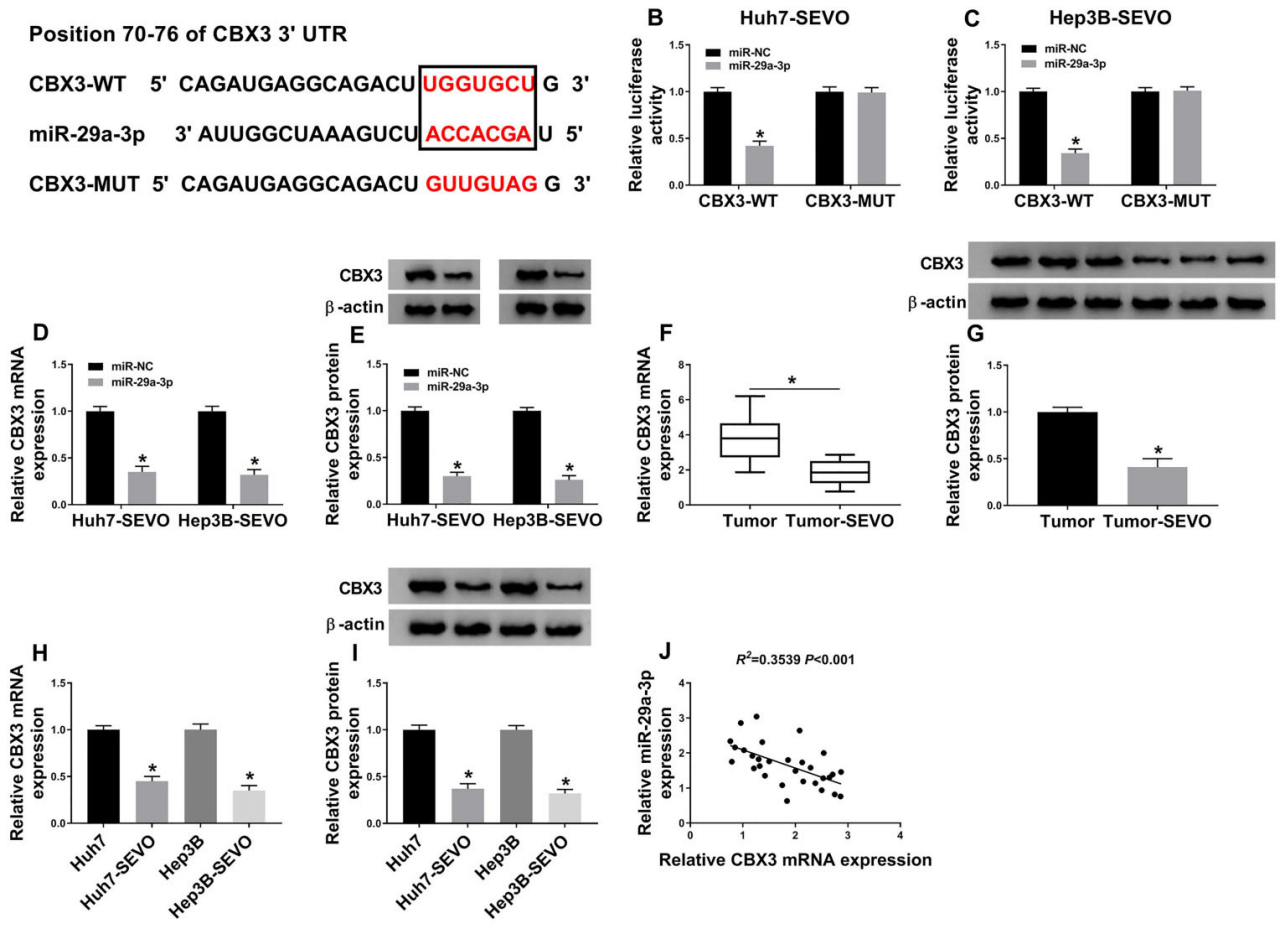
miR-29a-3p targeted CBX3. **A**, The interaction between miR-29a-3p and CBX 3'UTR was predicted by online tool Targetscan. **B** and **C**, Huh7 and Hep3B cells were treated with sevoflurane (SEVO) for 48 h. The luciferase activity of Huh7-SEVO and Hep3B-SEVO cells transfected with CBX3-WT (wild type) or CBX3-MUT (mutant) and miR-29a-3p or miR-NC (negative control) was examined (n=3). **D** and **E**, The mRNA and protein levels of CBX3 were measured in Huh7-SEVO and Hep3B-SEVO cells transfected with miR-29a-3p or miR-NC (n=3). **F** and **G**, The mRNA and protein levels of CBX3 were analyzed in hepatocellular carcinoma (HCC) tumor tissues (n=30) and SEVO-treated HCC tumor tissues (n=30). In **F**, data are reported as median and interquartile range. *P<0.05 (Kruskal-Wallis). **H** and **I**, The mRNA and protein levels of CBX3 were detected in Huh7 and Hep3B cells, as well as in Huh7-SEVO and Hep3B-SEVO cells (n=3). **J**, The association between miR-29a-3p and CBX3 levels was investigated. Data are reported as means±SD. *P<0.05 (Student's *t*-test or ANOVA).

The online tool Targetscan predicted that CBX3 was a potential target gene of miR-29a-3p (Figure 5A). Dual-luciferase reporter assay showed that the luciferase activity of the cells transfected with CBX3-WT and miR-29a-3p was suppressed, while there was no change in cells transfected with CBX3-MUT and miR-29a-3p (Figure 5B and C). Subsequently, the effect of miR-29a-3p on CBX3 expression was investigated. The results suggested that the mRNA level and protein level of CBX3 were significantly downregulated by miR-29a-3p overexpression (Figure 5D and E). In addition, decreased CBX3 mRNA and protein expressions were found in SEVO-treated HCC tissues (Figure 5F and G) and cells (Figure 5H and I). Furthermore, the CBX3 level was negatively correlated with miR-29a-3p level in SEVO-treated HCC tissues (Figure 5J). Overall, miR-29a-3p negatively regulated CBX3 expression.

### KCNQ1OT1 inhibited miR-29a-3p expression to increase CBX3 level

**Figure 6:**
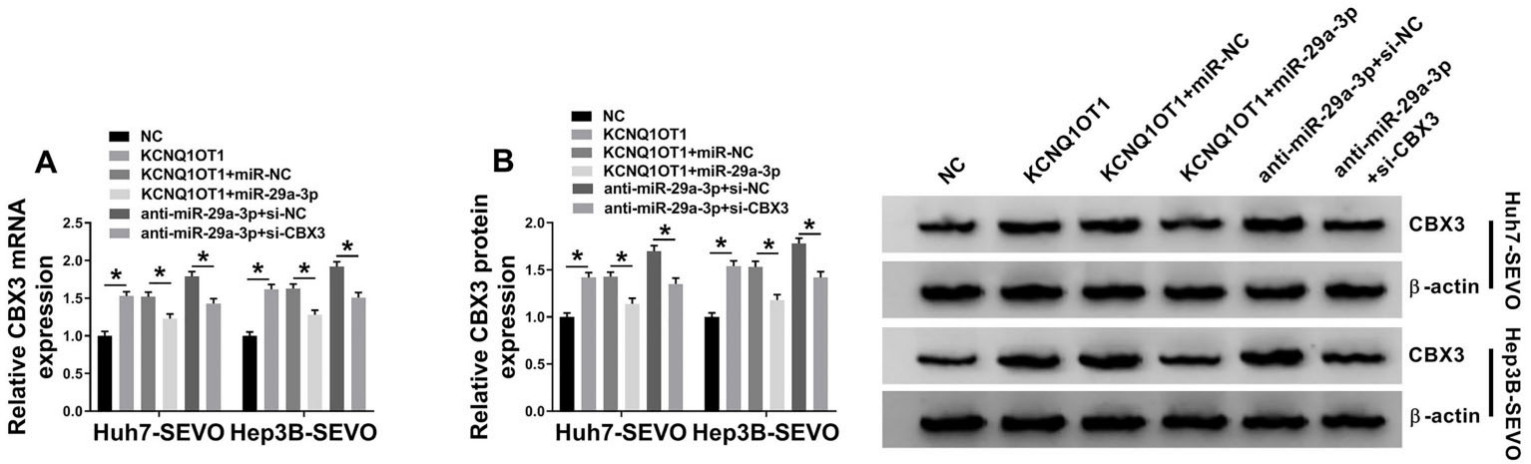
KCNQ1OT1 sponged miR-29a-3p to regulate CBX3 expression. Huh7 and Hep3B cells were treated with sevoflurane (SEVO) for 48 h. **A** and **B**, The level of CBX3 was detected in Huh7-SEVO and Hep3B-SEVO cells transfected with negative control (NC), KCNQ1OT1, KCNQ1OT1 + miR-NC, KCNQ1OT1 + miR-29a-3p, anti-miR-29a-3p + si-NC, and anti-miR-29a-3p + si-CBX3 (n=3). Data are reported as means±SD. *P<0.05 (Student's *t*-test or ANOVA).

To analyze the association among KCNQ1OT1, miR-29a-3p, and CBX3, Huh7-SEVO and Hep3B-SEVO cells were transfected with NC, KCNQ1OT1, KCNQ1OT1 + miR-NC, or KCNQ1OT1 + miR-29a-3p, respectively. Then, qRT-PCR assay and western blot assay were employed to detect the level of CBX3. As shown in Figure 6A and B, the mRNA and protein expressions of CBX3 were upregulated by KCNQ1OT1 overexpression or miR-29a-3p inhibitor, which were partly rescued by miR-29a-3p upregulation or CBX3 knockdown. These data revealed that KCNQ1OT1 upregulated CBX3 level via inhibiting miR-29a-3p expression.

### KCNQ1OT1 regulated HCC progression through the miR-29a-3p/CBX3 axis in SEVO-treated HCC cells

**Figure 7:**
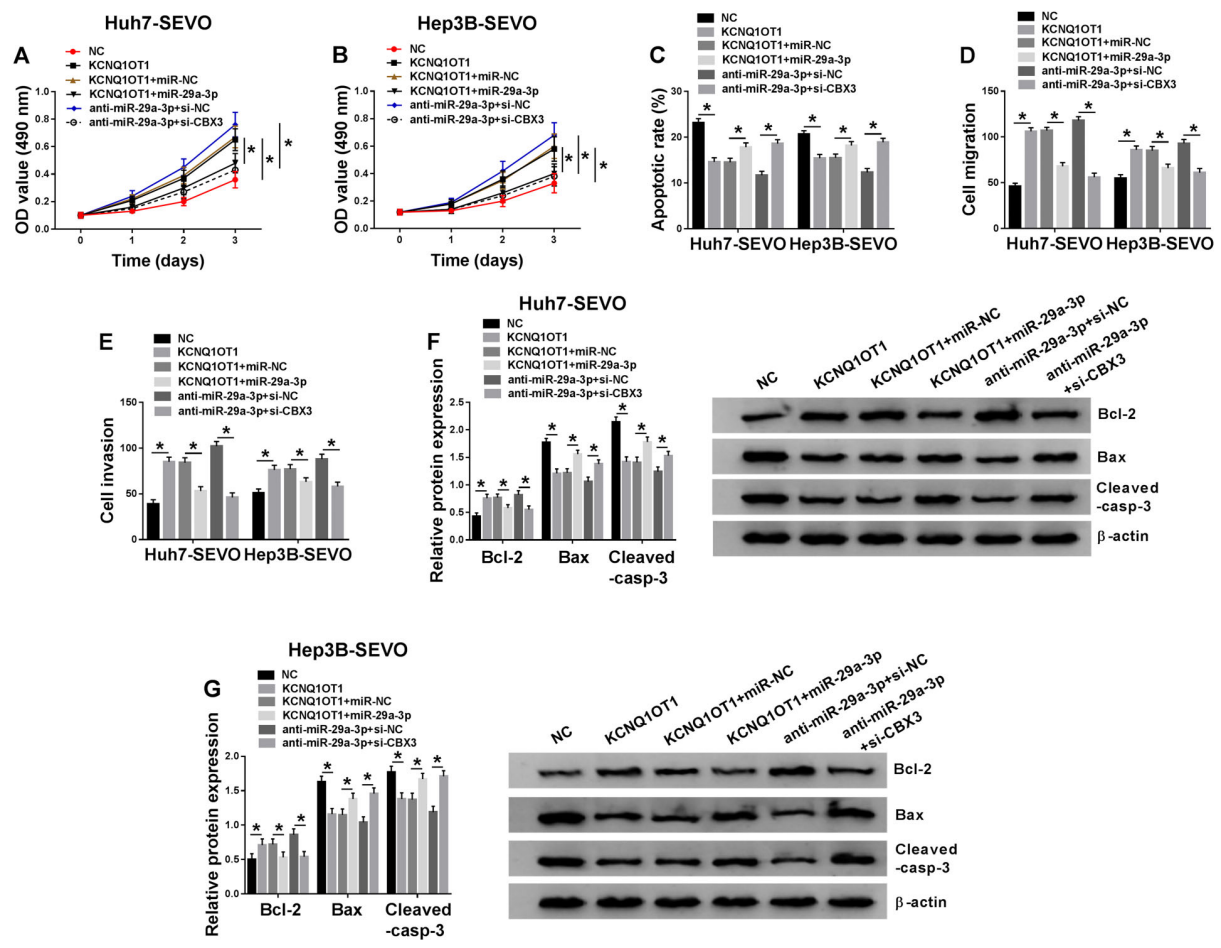
KCNQ1OT1 regulated hepatocellular carcinoma progression by sponging miR-29a-3p to regulate CBX3. Huh7 and Hep3B cells were treated with sevoflurane (SEVO) for 48 h. **A** and **B**, MTT assay was conducted to measure cell proliferation (n=3). **C**, Flow cytometry was employed to examine cell apoptosis rate (n=3). **D** and **E**, Cell migration and invasion were investigated using transwell assay (n=3). **F** and **G**, Western blot assay was performed to determine the levels of cell apoptosis-related proteins (n=3). Huh7-SEVO and Hep3B-SEVO cells were transfected with negative control (NC), KCNQ1OT1, KCNQ1OT1 + miR-NC, KCNQ1OT1 + miR-29a-3p, anti-miR-29a-3p + si-NC, and anti-miR-29a-3p + si-CBX3. Data are reported as means±SD. *P<0.05 (Student's *t*-test or ANOVA).

As demonstrated in Figure 7A and B, KCNQ1OT1 overexpression promoted cell proliferation in SEVO-treated HCC cells, whereas this action was weakened by the upregulation of miR-29a-3p. Cell apoptosis was suppressed by KCNQ1OT1 overexpression in SEVO-treated HCC cells, and then partly rescued by upregulation of miR-29a-3p (Figure 7C). Moreover, we found that upregulation of miR-29a-3p reversed the effect of KCNQ1OT1 overexpression on cell migration and invasion (Figure 7D and E) and the effect of KCNQ1OT1 overexpression on the levels of Bcl-2, Bax and Cleaved-casp-3 was weakened by upregulation of miR-29a-3p (Figure 7F and G). The results in Figure 4 suggested that the inhibition effect of SEVO HCC progression was reversed by miR-29a-3p inhibitor. Here, we confirmed that CBX3 depletion blocked the effects of miR-29a-3p inhibitor on cell proliferation, apoptosis, migration, and invasion in SEVO-treated HCC cells (Figure 7A-G). Taken together, KCNQ1OT1 mediated cell proliferation, apoptosis, migration, and invasion in SEVO-treated HCC cells by regulating the miR-29a-3p/CBX3 axis.

## Discussion

In recent years, SEVO has been used for the treatment of human cancers, including HCC (19,20). lncRNAs were identified to exert crucial roles in HCC development (21,22). For instance, Zhao et al. (23) suggested that lncRNA AWPPH enhanced the proliferation and mobility of HCC cells through targeting Y-box-binding protein 1 (YBX1). Guo et al. (24) demonstrated that lncRNA small nucleolar RNA host gene 16 (SNHG16) was highly expressed in HCC tissues and cells, as well as SNHG16 depletion suppressed cell growth and drug resistance in HCC cells. Li et al. (25) confirmed that lncRNA ARSR elevated cell doxorubicin resistance by regulating protein kinase B (AKT) pathway in HCC. KCNQ1OT1, an oncogene, has been shown to promote the growth of cancer cells, including HCC (26). However, the role of KCNQ1OT1 SEVO-regulated HCC development is unclear. In this study, we revealed that SEVO repressed proliferation and mobility, and accelerated apoptosis of HCC cells. Furthermore, the inhibitory effect of SEVO on HCC progression was weakened by overexpression of KCNQ1OT1. In addition, we confirmed that KCNQ1OT1 level was downregulated by SEVO treatment in HCC tissues and cells. These results demonstrated that SEVO suppressed HCC progression by regulating KCNQ1OT1 expression.

Amounting evidence suggests that lncRNAs could act as sponges for miRNA to regulate its expression in human cancers (27). For example, SNHG16 targeted miR-340 to regulate the progression of osteosarcoma (28). We then used the online tool Starbase to predict the potential target genes of KCNQ1OT1, and found that miR-29a-3p harbored the binding sites with KCNQ1OT1. Furthermore, KCNQ1OT1 negatively regulated miR-29a-3p level in SEVO-treated HCC cells. As a tumor suppressor, miR-29a-3p repressed cell growth in a variety of cancers, such as colorectal carcinoma (29), human laryngocarcinoma (30), gastric cancer (31), and HCC (32). More importantly, miR-29a was involved in the antitumor activity of SEVO in HCC (14). Consistent with these data, our results indicated that miR-29a-3p knockdown weakened the inhibitory effects of SEVO on HCC progression. Moreover, miR-29a-3p was upregulated in SEVO-treated HCC tissues and cells. Thus, it was speculated that SEVO suppressed HCC progression through regulating the KCNQ1OT1/miR-29a-3p axis. This hypothesis was then confirmed by our experiments, which suggested that upregulation of miR-29a-3p weakened the effect of KCNQ1OT1 upregulation on the growth of SEVO-treated HCC cells. Previous studies suggested that CBX3 promoted the growth of HCC cells (18). The online tool Targetscan was used to seek the downstream genes of miR-29a-3p, and we found that CBX3 was a potential target of miR-29a-3p. Moreover, we found that miR-29a-3p interacted with CBX3 and negatively regulated CBX3 expression. Furthermore, we found that CBX3 level was reduced by the treatment of SEVO in HCC tissues and cells. Our data revealed that miR-29a-3p regulated SEVO-treated HCC progression through repressing CBX3 expression. Moreover, we confirmed that KCNQ1OT1 suppressed the expression of miR-29a-3p to upregulate CBX3 level in SEVO-treated HCC cells. In conclusion, our findings demonstrated that KCNQ1OT1 weakened the antitumor effects of SEVO on HCC progression through modulating the miR-29a-3p/CBX3 axis, providing a potential target for the therapy of HCC patients.
